# Hell's BELs: Bacterial E3 Ligases That Exploit the Eukaryotic Ubiquitin Machinery

**DOI:** 10.1371/journal.ppat.1004255

**Published:** 2014-08-14

**Authors:** Jon Huibregtse, John R. Rohde

**Affiliations:** 1 Department of Molecular Biosciences, Institute for Cellular and Molecular Biology, The University of Texas at Austin, Austin, Texas, United States of America; 2 Department of Microbiology & Immunology, Dalhousie University, Halifax, Nova Scotia, Canada; University of North Carolina at Chapel Hill School of Medicine, United States of America

## How Do E3s Work in Eukaryotic Cells?

A common post-translational modification in eukaryotes is the covalent attachment of ubiquitin, a 76 amino acid protein, to specific proteins. Most commonly, ubiquitin is conjugated to ε–amino groups of lysine residues, and in unusual cases it can be conjugated to serine and cysteine residues or the terminal amino group of a protein. This process, referred to as ubiquitination (or ubiquitylation), can result in a variety of outcomes for a protein, depending upon how many Ub molecules are attached, whether a polyubiquitin chain is formed, and the nature of the chain. Mono-ubiquitination can result in relocalization of proteins, while most polyubiquitin chains (e.g., K48 and K11-linked chains) direct proteins for proteasomal degradation. Linkages of Ub formed using Lys 63 or by end-to-end linkages (also known as Met Ub) [1] are not directed to the proteasome and can mediate protein trafficking, scaffolding of protein complexes, or enzyme activation. Ub chains are also used for targeting invading microbes for clearance via xenophagy [2].

Ubiquitination is carried out by a series of enzymes. First, a ubiquitin activating enzyme (E1) forms a thioester with the C-terminus of Ub. The activated Ub is then transferred to one of many (∼40 human) ubiquitin conjugating enzymes (E2s). Finally, the E3 enzymes (perhaps over 500 human E3s) direct the transfer of Ub to specific substrates. In eukaryotic cells there are two general classes of E3 ubiquitin ligases. The HECT (Homologous to E6-AP Carboxyl Terminus) and Ring Between Ring (RBR) domain E3s possess an invariant catalytic Cys residue that accepts Ub from a charged E2 before catalyzing transfer of Ub to substrates. Other E3s contain a Really Interesting New Gene (RING) or RING-like domain (U-box) that recruits a charged E2, as well as a domain that recruits substrates. Ub is then transferred from the E2 to the substrate, with the E3 serving primarily as a scaffold. Some RING E3s are single polypeptides, while the cullin-RING Ligases (CRLs) are modular multisubunit complexes [3]. Mammalian CRLs are nucleated by one of seven cullin family members, with a RING domain protein that binds to its C-terminus. The N-terminal region of the cullin binds specific cullin adaptor proteins that engage substrate receptor proteins; the most studied class of substrate receptors are the F-Box proteins. CRLs are subject to an additional level of control by a ubiquitin-like modifier protein, Nedd8.

E3 enzymes have two important roles. First, they recognize substrates and position them for ubiquitination. Second, E3s dictate the nature of the Ub linkage(s), which will determine the substrate's fate. For HECT E3s, the Ub chain type is dictated by the C-terminal lobe of the HECT domain [4]. By contrast, RING-type E3s direct Ub chain type based upon the charged E2 that they recruit [5].

**Figure 1:**
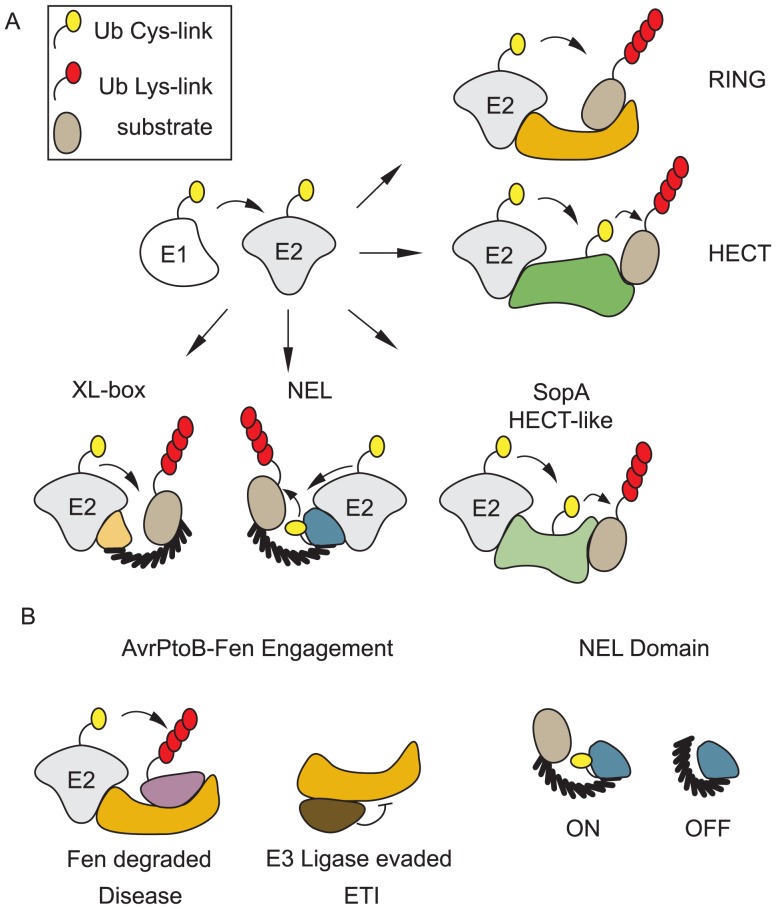
Bacteria encode E3 ubiquitin ligases of many classes. BELs share little or no sequence homology with eukaryotic E3s; however, structural similarities exist between BELs and eukaryotic E3s. Some BELs display entirely novel interactions with their cognate E2s. **A.** There are examples of bacterial E3 ligases that function similar to RING-type or HECT E3s in eukaryotic cells. Some BELs have novel mechanisms of ubiquitin transfer and interact with E2s in unique ways. **B.** Evidence exists for regulation of BEL activity inside eukaryotic cells. NEL domain enzymes are autoinhibited until they engage their substrates. AvrPtoB, a RING-type E3, can promote disease by ubiquitinating and causing degradation of the R proteins Pto and Fen (purple). Pto (brown) can also associate with AvrPtoB through an alternate domain allowing it to evade ubiquitination and initiating an ETI.

Successful pathogens use proteins that interfere with host cell function that are delivered into eukaryotic host cells via specialized secretion systems and collectively referred to as “effectors.” Remarkably, although ubiquitin is restricted to eukaryotic cells, the past decade has revealed that both bacterial and viral pathogens use effectors to interfere with or manipulate the ubiquitination system [6]. This involves a large number of Bacterially encoded E3 ubiquitin Ligases (BELs). There are multiple RING-type BELs, HECT-like BELs, and even BELs that bear no resemblance to known eukaryotic ubiquitin ligases (Figure 1A).

## BELs That RING

The first identification of a bacterially encoded E3 ubiquitin ligase was AvrPtoB from the plant pathogen *Pseudomonas syringae* pv. *tomato* (*Pst*) [7]. Plants use resistance proteins (R proteins) that recognize effectors from would-be pathogens. When R proteins engage bacterial effectors, they initiate an Effector Triggered Immune (ETI) response that prevents systemic disease [8]. AvrPtoB effectors from some *P. syringae* strains are recognized by the R protein Fen that initiates ETI. Although *Pst* AvrPtoB shared no sequence similarity to proteins of known function, the crystal structure revealed striking structural conservation with RING E3s [9]. AvrPtoB was shown to possess E3 ligase activity in vitro and to suppress ETI, demonstrating a role in pathogenesis [7]. AvrPtoB disrupts ETI by targeting Fen and Pto kinases for ubiquitination [10].

Bioinformatic approaches revealed that *Legionella pneumophila* encodes a number of F-box containing proteins [11]. Studies have demonstrated that these F-Box proteins function in infected cells as components of CRLs. In this case, BEL activity generates free amino acids that serve as fuel for *Legionella* growth inside infected cells [12]. *Legionella* encodes a BEL, LubX, that contains multiple U-boxes [13]. LubX targets another *Legionella* effector, the kinase SidH, for destruction, establishing LubX as a “metaeffector” that may act to coordinate spatiotemporal control of the effector repertoire within the host cell [14]. Thus, the targets of BELs should not necessarily be assumed to be host-encoded proteins.

One abundant class of RING-type E3s is the NleG family, encoded in enterohaemorrhagic *Escherichia coli* and *Citrobacter rodentium* [15]. NleGs were identified using NMR structural studies. Although no sequence similarity exists, the NleGs show close structural similarity to RING-type U-box proteins. As yet, no phenotypes have been associated with *nleG* mutants, but their large number points toward an important role.

## HECT-Like BELs

Discovery of HECT-like BELs followed a similar path as did AvrPtoB and NleG, driven in part by structural biology. The closely related effectors SopA, from *Salmonella enterica*, and NleL, from enterpathogenic *E. coli*, were both known to play a role in dampening host inflammation upon infection. While these effectors share no sequence homology to proteins of known function, Zhou and coworkers demonstrated that SopA functioned as a HECT-like enzyme [16]. The mechanisms of ubiquitin transfer had an absolute requirement for a catalytic Cys residue, and SopA was shown to form a Cys∼Ub thioester intermediate. These data suggest a mechanism of Ub transfer similar in mechanism to that of HECT enzymes, but it is clear that there will be significant differences as well. First, the proposed substrate-binding domain is adjacent to the E2 binding site, in contrast to what has been proposed for eukaryotic HECTs [17]. Secondly, SopA and NleL interact with the same region of Ubc8 as do mammalian HECT or RING domain E3s, but differ in the precise E2 residues that are required for BEL activity [18].

## BELs That Are NELs—And More…

*Shigella* spp. use T3SS effectors to cause shigellosis. The most abundant effectors produced by *Shigella* upon contact with human cells are the IpaH proteins, a class of proteins that exist in many gram-negative pathogens of animals and plants [19]. Their N-terminal domain consists of a series of leucine rich repeat (LRR) domains that share high similarity with YopM from *Yersinia* spp. [20]. The C-terminal domain is highly conserved among IpaH family members. Using yeast as a surrogate genetic system, IpaH9.8 was shown to possess E3 ubiquitin ligase activity and destroy the MAPKK Ste7 in a proteasome-dependent manner [19]. The IpaH family member SspH1 from *Salmonella* was shown to also be an E3 ligase that could ubiquitinate a known mammalian interacting protein, PKN1, in vitro [19]. Earlier studies had already shown that substrate specificity was dictated by the LRR domains [21]. The ability to ubiquitinate substrates was shown to rely on a Cys residue that is invariant among the more than 50 proteins that comprise the IpaH family [19]. Again, the IpaH family members shared no sequence similarity to proteins of known function. The crystal structure was solved independently by three groups and revealed that the catalytic domain was entirely alpha helical, had no resemblance to other E3 enzymes, and was coined the NEL domain (novel E3 ligase) [22]–[24]. Similar to HECTs, NEL domains use the invariant Cys residue to form a thioester linkage with Ub [22], [23]. Mutations in E2 enzymes that ablate activity towards RING or HECT domain E3s were shown to be fully functional for Ub transfer to NELs, suggesting a novel E2–E3 interaction [22]. Elegant structural studies showed that NEL domain BELs recognize E2∼Ub conjugates (activated E2s) and use a region of E2s that had not before been implicated in Ub transfer. Based on these studies, a radically new “see-saw” mechanism for Ub transfer was proposed for NEL-domain BELs [25].

The most recent addition to the BEL family is the XL-box domain BEL, XopL, from *Xanthomonas campestris* pv. *vesicatoria* (*Xcv*), a pathogen of tomatoes and peppers [26]. The general architecture of XopL is similar to NEL domain E3s in that it contains an N-terminal domain of LRRs with homology to those of NEL LRRs. The C-terminal domain is a novel E3 ubiquitin ligase domain known as the XL-box. The LRR domain alone is required for suppressing ETI; however, the catalytic domain is required to cause disease in the plant [26]. The XL domain lacks Cys residues, suggesting they function in a mechanism similar to RING-type E3s. Some E2 residues that are absolutely required for RING and HECT function were also required for XopL function, but others were not [26].

## Is BEL Activity Regulated?

The activity of NEL domain E3s is negatively regulated by the LRR domain in the absence of their substrates, presumably to prevent premature autoubiquitination until they can productively engage their substrates [22], [23], [27]. The first NEL domain enzyme–substrate structure has been characterized [28]. The LRR of SspH1 binds the HR1b coiled-coil subdomain of PKN1. This report provides the first direct evidence that substrate engagement activates the catalytic activity of NELs. In this case, a straightforward “displacement model” between a linear PKN1 motif and the inhibitory residues within the NEL domain compete for binding to residues within the LRR [28]. As LRR domains are remarkably diverse scaffolds for protein–protein interactions, it remains to be tested if the SspH1–PKN1 paradigm will emerge as a universal mechanism for effector–substrate recognition and activation for BELs. Precise BEL–substrate interactions were recently shown to effect distinct outcomes in the Pst system. The R proteins Fen and Pto interact near the Ring domain of AvrPtoB, resulting in their ubiquitination and degradation [10]. Pto can also interact with an AvrPtoB domain distal to the Ring domain [29]. Binding at the distal domain allows Pto to evade ubiquitination by the AvrPtoB E3 ligase and to activate an ETI response.

Though they do not have a ubiquitin system, bacteria encode a wide variety of E3 ubiquitin ligases that are delivered into the host cells that they infect using specialized secretion systems. A trend among BELs is that while they possess little to no sequence homology with eukaryotic E3s, they often share structural similarity. Notable exceptions are NEL domain and XL-box BELs, suggesting the idea that there are structurally related NEL domain enzymes encoded by eukaryotes. Determining the spectrum of BEL substrates in their respective hosts is an achievable goal. The identification of the eukaryotic proteins that BELs target will increase our understanding of immune functions and provide insights to help combat infection.
